# Heavy Metal Distribution in Aquatic Products from Eastern Guangdong and Associated Health Risk Assessment

**DOI:** 10.3390/toxics12120881

**Published:** 2024-12-02

**Authors:** Jinyan Liu, You’an Yu, Zewei Sun, Keqin Zhang, Ping Li, Wenhua Liu, Ran Bi

**Affiliations:** 1 Guangdong Provincial Key Laboratory of Marine Disaster Prediction and Prevention, and Institute of Marine Sciences, Shantou University, Shantou 515063, China; liujy@stu.edu.cn (J.L.); swift0828@163.com (Y.Y.); kqzhang@stu.edu.cn (K.Z.); liping@stu.edu.cn (P.L.); whliu@stu.edu.cn (W.L.); rbi@stu.edu.cn (R.B.); 2 Institute of Marine Sciences, Shantou University, Shantou 515063, China; 3 Guangdong Engineering Technology Research Center of Offshore Environmental Pollution Control, Shantou 515063, China

**Keywords:** marine pollution, heavy metal, aquatic products, health risk

## Abstract

With the rapid industrialization and urbanization of coastal areas, marine pollution (such as heavy metals) is increasingly contaminating the environment, posing significant public health risks. Eastern Guangdong, a key aquaculture and fisheries hub in China, has a growing market for aquatic products. Heavy metals persist in the environment and are difficult to degrade and bioaccumulate in marine organisms through the food web, presenting carcinogenic and mutagenic risks to humans, as top predators. This study analyzed 10 key species commonly consumed by residents of eastern Guangdong (bivalves, crustaceans, and fish), measuring the concentrations of six heavy metals (As, Cd, Cr, Cu, Ni, Pb) using inductively coupled plasma mass spectrometry (ICP-MS). Pollution levels were assessed using the pollution index (Pi), and dietary exposure risks were evaluated via the target hazard quotient (THQ) for different age groups. Results showed that Pi values for all metals were within normal background levels, but bivalves had a high capacity for Cd accumulation, with pollution severity ranking as bivalves > crustaceans > fish. The THQ values for both adults and teenagers were <1 across all samples, indicating no risk to the health of residents. However, the TTHQ for *Sanguinolaria* sp. exceeded 1, indicating potential health risks. This study highlights the health risks of consuming heavy metal-contaminated aquatic products, particularly bivalves. Reducing the consumption of these high-metal species could help lower dietary exposure and associated risks. Our findings provide essential data for the quality assessment of aquatic products and offer dietary recommendations for residents in eastern Guangdong.

## 1. Introduction

In recent decades, due to their nutritional value and high-quality protein, the global consumption of aquatic products has increased rapidly [1,2,3,4]. However, it is well known that aquatic products can accumulate heavy metals from the environment, which then move up the food chain, posing potential health risks to humans when contaminated products are consumed [5,6,7,8]. Heavy metal contamination in aquatic products is a global issue, with associated health risks from aquatic products and heavy metal intake being a concern worldwide [9,10,11]. Overall, the heavy metal pollution status of aquatic products abroad is similar to that in China [12,13,14], with fish species particularly affected by As contamination, especially common large fish like tuna and salmon. Bivalves primarily face Cd and As pollution. For example, Loaiza et al. [15] found that in aquatic products along the coast of Peru, approximately 20% of bivalves and about 30% of crustaceans had As levels exceeding the maximum residue limits for human consumption.

Heavy metals in the marine environment originate from both natural and anthropogenic sources [16,17]. Natural sources include undersea volcanic eruptions, weathering of crustal rocks, soil erosion, and atmospheric deposition, which establish the baseline levels of heavy metals in oceans [18]. Human activities, however, are significant contributors to marine heavy metal pollution [19]. Key sources include smelting and electroplating (releasing As, Cd, Cr, Cu, and Ni), metal-based manufacturing industries (involving As, Cd, Cu, and Ni), fossil fuel combustion (primarily Pb), and the use of pesticides and insecticides (introducing As, Pb, Cd, and Cu) [20]. These pollutants are discharged into the marine environment through wastewater, exhaust gases, and waste residues, serving as primary contributors to marine heavy metal contamination [18,21,22]. Existing studies have shown that heavy metal bioaccumulation concentrations vary significantly among different aquatic products. These differences in accumulation may be closely related to the habitats, feeding behaviors, and trophic levels of the aquatic products [23,24].

Eastern Guangdong, a major region for aquaculture and fisheries in China, offers a wide variety of aquatic products, including fish, bivalves, and crustaceans [25,26]. In addition, the economic prosperity and high population density of the eastern Guangdong region result in frequent consumption of aquatic products as a staple in the local diet, serving as a substantial source of essential nutrients and high-quality protein [27]. However, with the intensification of coastal industrialization and urbanization, the marine environment in eastern Guangdong is facing challenges from heavy metal pollution [28]. Marine organisms absorb and bioaccumulate heavy metals, such as As, Cd, and Pb, through the food web [28,29,30]. These heavy metals gradually concentrate through the food chain, ultimately reaching humans, who are at the top of the food chain [31]. Due to the high frequency of aquatic product consumption, the residents of eastern Guangdong may experience an increase in heavy metal exposure levels from consuming contaminated aquatic products, potentially leading to health risks such as carcinogenic, mutagenic effects, and damage to the nervous and immune systems [32,33].

This project conducted a questionnaire survey to identify the primary aquatic product types consumed by residents of eastern Guangdong. By analyzing the heavy metal content in commonly consumed aquatic products and cross-referencing with national food safety standards on contaminants and aquatic product consumption frequency, this study assesses health risks for local residents. Findings will provide data to support quality assurance of aquatic products sold in eastern Guangdong and offer dietary recommendations to its residents.

## 2. Materials and Methods

### 2.1. Study Site and Sample Collection

Eastern Guangdong is one of the most developed industrial regions in China and a global manufacturing hub [34]. With the continuous development of the Pearl River Delta, economic growth has led to the release of substantial amounts of heavy metals into coastal waters, severely contaminating local sediments and water bodies and resulting in heavy metal pollution in aquatic products [35,36]. Previous studies have reported instances of heavy metal contamination in marine organisms in this region [37,38,39]. In this study, all aquatic products were purchased from local markets in Shantou, with sample collection taking place in January 2021. The ten commonly consumed species included *Mugil cephalus*, *Pampus argenteus*, *Larimichthys crocea*, *Acanthopagrus latus*, *Siganus fuscescens*, *Penaeus vannamei*, *Scylla* sp., *Ostreidae* sp., *Sanguinolaria* sp., and *Mytilus eduli*. After purchasing, the samples were transported to the laboratory, weighed, and dissected into muscle, gill, and liver tissues. All tissues were homogenized using a meat grinder and stored at −20 °C for subsequent analysis.

### 2.2. Sample Analysis

The samples were taken from representative edible parts, gills, and livers using sterilized stainless steel scissors and tweezers, wrapped in aluminum foil, and stored in plastic ziplock bags at −20 °C for freezing. After all samples were processed, they were placed in a −80 °C ultra-low temperature freezer for pre-cooling for 24 h, followed by freeze-drying for 96 h. Once dried, the samples were ground into a fine powder using an agate mortar for later use. A weight of 0.10 g ± 0.01 g (for samples with a smaller total amount, 0.05 g ± 0.005 g) of the processed samples was placed into a digestion tube, to which 3 mL of 65% HNO_3_ (trace metal grade, Thermo Fisher Scientific Inc., Waltham, MA, USA) and 1 mL of 30% H_2_O_2_ (Merck KGaA, Darmstadt, Germany) were added. The mixture was allowed to stand in a Teflon^®^-lined digestion vessel until no bubbles were produced and then placed into a microwave digestion extraction instrument to ensure complete decomposition of the samples. The Mars Xpress™ (CEM, Wilson, NC, USA) digestion program was set to heat up over 20 min to reach 190 °C and maintained at that temperature for 20 min. Once cooled to room temperature, the solution was transferred to a 50 mL centrifuge tube and diluted to 50 mL with Milli-Q^®^ water (Millipore Sigma, Burlington, MA, USA), with the addition of internal standard solutions of (In (GSB04-1731-2004) and Rh (GSB04-1746-2004), National Institute of Metrology, Beijing, China), achieving a final concentration of 10 μg/L for the internal standard elements. The concentration of As, Cd, Cr, Cu, Ni, and Pb was determined using ICP-MS (iCAP TQ Thermo Fisher, Weil am Rhein, Germany) under the O_2_ mode (Table S1). The recovery efficiency of six metals was determined to be between 83.58% and 102.86%.

The inorganic arsenic fraction in the aquatic products was determined using HPLC (Ultimate 3000, Thermo Fisher Scientific Inc., Waltham, MA, USA) coupled with ICP-MS (iCAP TQ, Thermo Fisher Scientific Inc., Waltham, MA, USA) under O_2_ mode (Table S1). In each sample extract, 0.1 mL of 30% H₂O₂ solution was added to facilitate the oxidation reaction of As^III^, converting it into As^V^. This treatment method allows us to accurately quantify the concentration of As^V^, thereby enabling the calculation of the total inorganic arsenic (iAs) in the sample, which includes the sum of As^III^ and As^V^. This process is crucial for assessing the contamination levels of inorganic arsenic in aquatic products and helps us better understand their potential environmental and health risks. External calibrations were performed using iAs^III^ (GBW08666) and iAs^V^ (GBW08667), acquired from NIM (National Institute of Metrology, Beijing, China).

### 2.3. Quality Assurance and Quality Control (QA/QC)

All samples were thoroughly washed three times with Milli-Q water (18 MΩ·cm) prior to digestion to ensure the removal of external debris and contaminants. To avoid plastic and fiber contamination, all containers and tools used were made of non-plastic materials. Glassware was wrapped in aluminum foil and treated at 450 °C for 4 h before use to remove any potential organic substances. Before use, all tools were rinsed three times with Milli-Q water to eliminate any residual contaminants. During the experiment, all materials were immediately covered with aluminum foil when not in use to prevent external contamination. In each sample preparation batch, procedural blank samples (*n* = 5) were processed. These blank samples did not contain any tissue, effectively identifying and correcting potential contamination from reagents or the laboratory environment. Certified reference materials GBW 10,024 (National Institute of Metrology, Beijing, China), SRM 1566b (National Institute of Standards and Technology), tuna fish tissue BCR-627 (European Commission’s Joint Research Centre), and trace elements and arsenic compounds in seaweed (NMIJ-7405b, National Metrology Institute of Japan) were used for comparative analysis to ensure the accuracy and comparability of the test results. The limits of detection (LOD), the limit of quantification (LOQ), and detected certified reference material for heavy metals including inorganic arsenic are shown in Tables S2–S6.

### 2.4. Questionnaire Design

The project involved designing and distributing a questionnaire through Questionnaire Star, with a total of 231 responses collected. The respondents mainly included teenagers, men, women, and the elderly, all of whom were permanent residents of the eastern Guangdong region. Due to seasonal limitations, we selected ten commonly consumed aquatic products in the eastern Guangdong area for analysis (*Mugil cephalus*, *Pampus argenteus*, *Larimichthys crocea*, *Acanthopagrus latus*, *Siganus fuscescens*, *Penaeus vannamei*, *Scylla* sp., *Ostreidae* sp., *Sanguinolaria* sp., *Mytilus eduli*). The questionnaire was designed to first ask respondents about their gender, age, and occupation to ensure the credibility of the survey results. Then, it inquired about the frequency of consuming commonly found aquatic products in the eastern Guangdong region. By collecting and analyzing the questionnaire data, we were able to determine the consumption frequency of different aquatic species in eastern Guangdong, as shown in Table 1.

### 2.5. Heavy Metal Pollution and Health Risk Assessment

This study employed the single-factor pollution index method (Pi) to calculate and assess the pollution levels of As, Cd, Cr, Cu, Ni, and Pb in different types of aquatic products based on the single-factor pollution index method and evaluation standards [40]. The specific formula is as follows:

(1) $$\mathrm{Pi}=\frac{\mathrm{Ci}}{\mathrm{Si}}$$

In the formula, Pi represents the single factor pollution index for the heavy metal, Ci is the concentration of heavy metal, mg/kg; Si is the maximum allowable limit for heavy metal as specified in GB 2762-2022 [41] “Food Safety Standards: Limits of Contaminants in Food”, mg/kg. Since there are currently no explicit standards for classifying heavy metal pollution levels in food or aquatic products in China, and existing standards differ significantly from those applicable to aquatic products, the classification criteria recommended in the literature were adopted for assessment. Specifically, when Pi < 0.2, it indicates a normal background value; when 0.2 ≤ Pi < 0.6, it indicates mild pollution; when 0.6 ≤ Pi < 1, it indicates mild pollution; when Pi ≥ 1, it indicates heavy pollution, meaning it exceeds the allowable limits.

This study employs the target hazard quotient (THQ) method to assess the health risks of As, Cd, Cr, Cu, Ni, and Pb in fish flesh for the exposed population. This method evaluates whether the contaminant doses in the food ingested by the exposed population exceed the reference dose by integrating various parameters, thereby determining the presence or absence of health risks. The specific formula is as follows:

(2) $$\mathrm{THQ}=\frac{\mathrm{EF}\;\times \;\mathrm{ED}\;\times \;\mathrm{FIR}\;\times \;C}{\mathrm{RDF}\;\times \;\mathrm{BW}\;\times \;\mathrm{TA}}\times 10^{-3}$$

In the formula: C is the concentration of heavy metals in fish samples, expressed in mg/kg. EF is the exposure frequency (365 days/year); ED is the exposure duration (70 years); FIR is the daily ingestion rate in g/capita/day (57.58 for fish, 6.00 for shrimp, and 5.54 for crab); the reference oral dose in mg × kgbw^−1^d^−1^ (3 × 10^−4^ for As, 1 × 10^−3^ for Cd, 1.5 for Cr, 4 × 10^−2^ for Cu, 2 × 10^−2^ for Ni, 4 × 10^−3^ for Pb); BW is the average body weight of individuals (45 kg for teenagers, 57.5 kg for men, 52.5 kg for women, and 51 kg for the elderly); TA is the average exposure time for non-carcinogenic sources (25,550 days). The evaluation criteria are as follows: THQ < 1 indicates a low impact on human health, while THQ ≥ 1, indicates a significant risk to human health [42]. The total THQ (TTHQ) of heavy metals for individual seafood is the sum of the following composition: TTHQ (individual seafood) = THQ (toxicant 1) + THQ (toxicant 2) + THQ (toxicant n) [43].

### 2.6. Data Analysis and Statistics

One-way analysis of variance (ANOVA) was performed in SPSS 22.0 (IBM, Armonk, NY, USA) to analyze the concentration of heavy metals in aquatic products, followed by Dunnett’s test. The normality and homogeneity of variances of the data were tested using the Shapiro–Wilk test and Levene’s test, respectively. Statistical analysis was conducted in SPSS 22.0 (IBM, USA). Spearman correlation analysis was used to explore the relationships between the concentrations of heavy metals in different tissues of fish. A significance level of *p* < 0.05 was considered statistically significant.

## 3. Results and Discussion

### 3.1. Accumulation of Heavy Metals in the Edible Tissues of Aquatic Products

**Figure 1:**
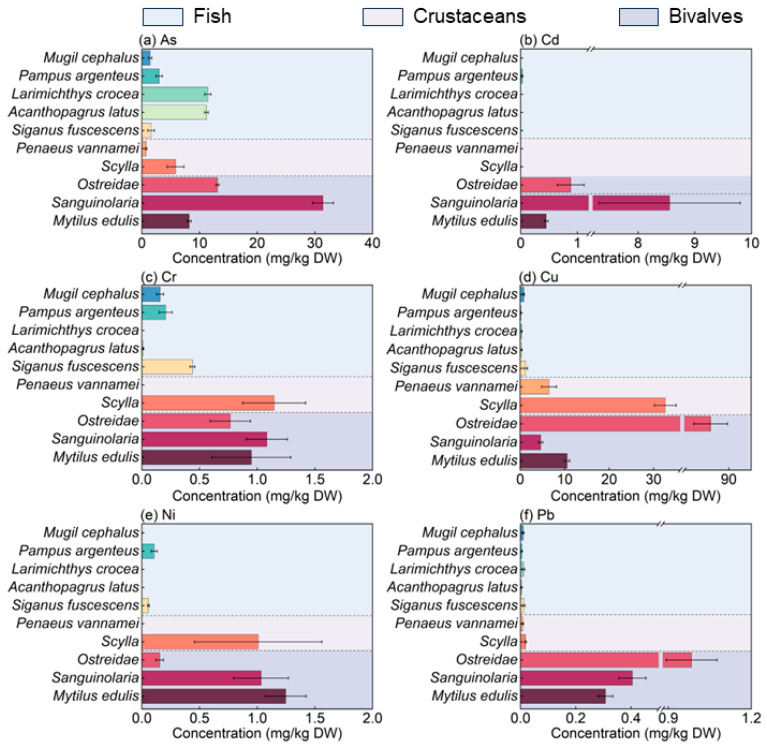
Heavy metal concentrations in the edible parts of aquatic products.

This study investigated the abundance and distribution of heavy metals in 10 aquatic products from the eastern Guangdong region. Heavy metals were detected in all aquatic products. Due to differences in heavy metal sources and the mechanisms of absorption and accumulation in organisms, the concentrations of heavy metals in aquatic products vary among different species. The concentrations of As, Cd, Cr, Cu, Ni, and Pb in the aquatic products ranged from 0.74 to 31.40 mg/kg, 0.002 to 8.56 mg/kg, 0.01 to 1.14 mg/kg, 0.14 to 85.93 mg/kg, 0.001 to 1.25 mg/kg, and 0.003 to 0.98 mg/kg, respectively (Figure 1). The concentrations of all heavy metals in aquatic products were highest in bivalves. The concentrations of As in fish, crustaceans, and bivalves exceeded the National Food Safety Standard of Limits for Contaminants in Food (GB 2762-2022) [41]. Saei-Dehkordi et al. [31] found that *Acanthopagrus latus* had a total arsenic content of 0.266 ± 0.040 mg/kg in winter, reflecting seasonal metabolic and habitat changes. Rumisha et al. [44] reported higher arsenic levels (3–6 mg/kg ww) in *Scylla* sp. from industrialized areas of western Tanzania. Additionally, Sloth et al. [45] found high concentrations of total arsenic in *Mytilus edulis* (up to 13.8 mg/kg ww), with 42% being inorganic arsenic, one of the highest reported in marine species. The concentrations of Cu in crustaceans and bivalves also exceeded this standard, suggesting the potential health risk via consumption. In comparison, the Cu concentration in *Scylla* sp. in this study (32.62 ± 2.47 mg/kg) was lower than that in crustaceans from Daya Bay, reported by Wang et al. [46] at 36.5 mg/kg. This result is consistent with the conclusions from the analysis of heavy metal concentrations in aquatic organisms from Taihu Lake, where shrimp demonstrated the highest Cu accumulation capacity [47]. Cu is an important trace element that is crucial for the growth and physiological functions of crustaceans and bivalves, which may explain why the Cu concentration found in *Ostreidae* sp. is relatively high (85.93 mg/kg). The Cd concentration in *Sanguinolaria* sp. among bivalves (8.55 ± 1.24 mg/kg) was higher than that reported in Tuolin Harbor in the eastern part of Guangdong Province (2.62 mg/kg) [38]. Cd is a non-essential toxic metal with no biological function, and its toxicity to humans occurs through biomagnification in the food chain. Relatively high Cd concentrations were found in bivalves, especially in *Ostreidae* sp. and *Sanguinolaria* sp., which were higher than values reported in all listed regions except for the coastal areas of Zhejiang. This finding aligns with previous research indicating that Cd is easily accumulated by bivalves [48,49]. This may be because bivalves are unable to effectively regulate Cd levels, leading to its accumulation in their bodies, and Cd may subsequently magnify through the food chain and accumulate in humans, warranting careful attention. In contrast, Cd concentrations in fish were lower, particularly in *Mugil cephalus* and *Larimichthys crocea*. The low Cd concentration detected in *Larimichthys crocea* from the Dachen Fishing Ground, as reported by Huang et al., [50] was consistent at 0.065 ± 0.001 mg/kg.

The pollution levels of heavy metals in the edible tissues of all ten aquatic product species tested are shown in Table 2. The concentrations of Cr and lead Pb were found to be within acceptable limits. Species such as *Mugil cephalus*, *Pampus argenteus*, *Siganus fuscescens*, and *Penaeus vannamei* exhibited low levels of heavy metals, with all heavy metals showing pollution indices (Pi values) within normal background ranges. However, the iAs concentration in fish and bivalves exceeds the acceptable limits; some individuals of *Sanguinolaria* sp. had Cd levels exceeding acceptable limits, while some *Scylla* sp. individuals reached low-level pollution for Ni. Additionally, both Cd and Cu concentrations in *Ostreidae* sp. reached low pollution levels. Bivalves exhibited higher concentrations of Cd, Cu, Ni, and Pb compared to fish and crustaceans, with only *Sanguinolaria* sp. having Cd levels that reached pollution thresholds. This aligns with the findings of Wang et al. [51] who identified bivalves as hyperaccumulators of Cd. This phenomenon is associated with various anthropogenic activities in the eastern Guangdong coastal waters. *Sanguinolaria* sp. mainly inhabits estuarine areas in the eastern Guangdong region, which receive the majority of pollutant discharges from industrial zones. Under turbid conditions, heavy metals (either in soluble form or adsorbed onto suspended particles) discharged from drainage outlets are filtered from the water column and subsequently accumulate in the soft tissues of bivalves. Research has indicated that bivalve mollusks from coastal provinces such as Shanghai and Fujian, as well as Zhanjiang in Guangdong, have shown signs of Cd pollution [52,53]. Notably, the Cd content in bivalve mollusks is consistently higher than that found in crustaceans and fish, indicating a strong bioaccumulation capacity for Cd in these organisms. The heavy metal content in fish tissues is lower than that in bivalves and crustaceans, which is within the range compared to other studies worldwide (Table S7) [50,54,55,56,57,58,59,60,61,62,63,64,65,66]. This is because metals are absorbed by fish through suspended particles, ion exchange during respiration, and the food chain, while bivalves inhabit sediments with higher metal concentrations [67]. This study highlights the need for continuous monitoring of heavy metal levels in aquatic products, particularly in coastal areas where industrial and agricultural activities may contribute to contamination. The elevated concentrations of heavy metals in bivalves could pose potential health risks to consumers, necessitating guidelines and regulations to ensure food safety. Furthermore, understanding the mechanisms behind heavy metal accumulation in bivalves can inform strategies for mitigating pollution in marine environments, protecting both aquatic ecosystems and public health.

### 3.2. Analysis of Heavy Metal Content in Fish Tissues (Muscle, Gill, Liver)

**Figure 2:**
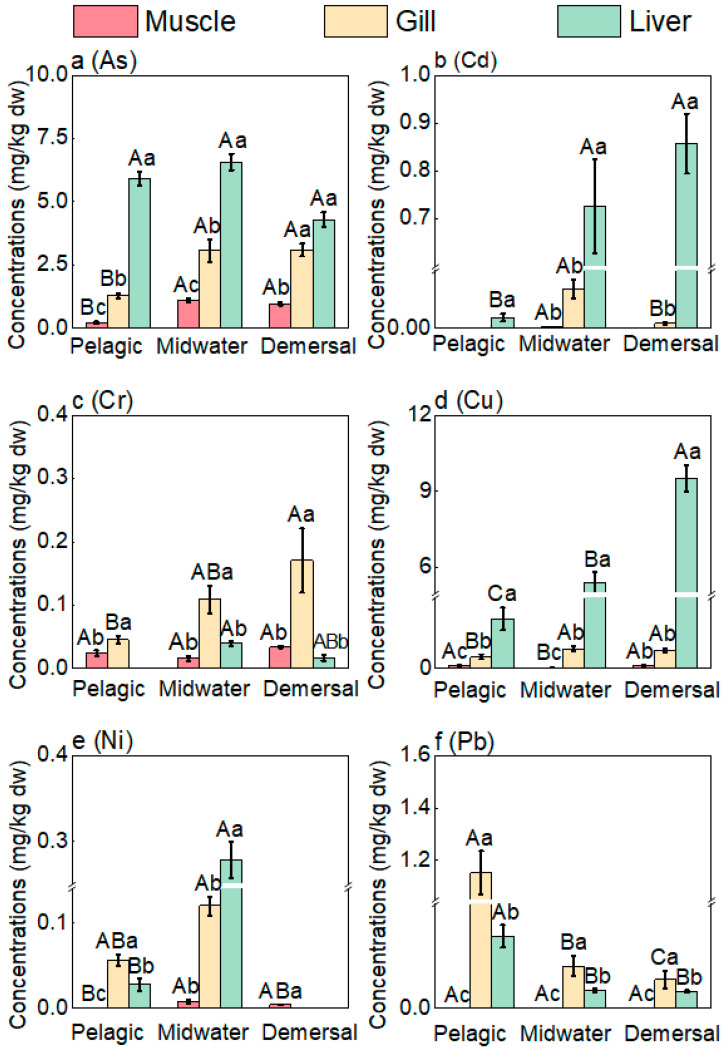
Heavy metal concentrations in fish muscle, gill, and liver. Capital letters indicate statistical significance among different habitats, while lowercase letters indicate statistical significance among different tissues (*p* < 0.05).

The accumulation capacity of heavy metals varies in different tissues of fish. The heavy metal content in the liver, gills, and muscle tissues of five fish species was analyzed. The overall concentrations of two heavy metals, As and Cu, followed the pattern of liver > gills > muscle, indicating that the primary accumulation site for these heavy metals in fish is the liver (Figure 2). The liver is the main organ for metabolism and detoxification [68,69]. Meanwhile, the liver is considered the primary organ for the metabolism and detoxification of any toxic substances, where they accumulate significantly [70,71,72]. During metabolic processes, heavy metals entering the body through the digestive tract are primarily detoxified in the liver and then transported through the bile ducts to redistribute to various tissues throughout the body [73]. This explains why the concentrations of As, and Cu are highest in the liver. The concentration of As in the gills was significantly higher than in the muscle. Similarly, the concentration of Cr in the gills of demersal fish was significantly higher than in the muscle, reaching 0.17 ± 0.05 mg/kg. Demersal fish are more likely to be exposed to heavy metals in sediments, which are considered the main source of heavy metals in marine fish [74,75]. The gills serve as the filtering and respiratory organs of fish, possessing a large surface area and direct contact with the external environment [76]. They filter and accumulate water pollutants and are involved in the excretion of metabolic products from the fish [77]. Consequently, the concentration of heavy metals in the gills is generally higher than that in the muscle.

In contrast, in midwater fish and demersal fish, the As concentration followed the pattern of liver > muscle > gills, with the As content in the liver of midwater fish being significantly higher than that in the muscle. Compared to published data, De Rosemond et al. [78] analyzed five freshwater fish species from Back Bay near Yellowknife in the Northwest Territories, Canada, reporting total arsenic concentrations in muscle ranging from 0.57 to 1.15 mg/kg and in liver from 0.42 to 2.25 mg/kg. We found that the As concentrations in the liver and muscle of fish were higher than those in the gills, which is consistent with the literature data [79,80]. Additionally, the larger liver may dilute the concentration of As in the liver.

Notably, in fish from different habitats, the overall concentrations of Cr and Pb in the gills followed the distribution pattern of gills > liver > muscle, with Pb concentrations in the gills ranging from 0.008 mg/kg to 0.298 mg/kg, while the muscle Pb concentration was below 0.002 mg/kg. Additionally, the Cr content in the gills of midwater fish was higher than in other tissues, reaching 0.17 ± 0.05 mg/kg. It can be inferred that different habitats may also result in variations in heavy metal accumulation in aquatic products. This is consistent with the findings of Ihunwo et al. [56] who observed that the metal concentration in the gills was higher than in the muscles, attributing this to the important functions of fish gills, such as gas exchange, ion transport, and nitrogen excretion. This suggests that the gills are the first organs to be exposed to waterborne pollutants like metals [81]. It is speculated that the primary source of Pb in fish is from the water adsorbed by the gill filaments, with only a very small amount of Pb transferred through the food chain, resulting in Pb accumulation primarily in the gills. Given that the concentration of heavy metals in the gills is higher than in the muscle, it is recommended that residents of eastern Guangdong change their cooking habit of not removing the gills when preparing fish.

### 3.3. Health Risk Assessment

Based on the average concentrations of various heavy metals in different types of aquatic products, the percentage of intake relative to the provisional tolerable weekly intake (PTWI) or tolerable upper intake level (UL) was assessed, with the results shown in Table 3. According to the survey data on per capita weekly aquatic product consumption (Table 1), “Multiple times per week” means three times a week, and “rarely consumed” means zero times per week. One month is considered as 4 weeks, with a serving size of 100 g per meal. The average weekly intake (AWI) of Cr and Cu among residents in eastern Guangdong accounted for a percentage of the UL (weekly), while the AWI of Cd and Pb accounted for a percentage of the PTWI (for adults), ranging from 0% to 14%, indicating that the amount of heavy metals ingested by adults in eastern Guangdong from commercially available aquatic products is far below the tolerable upper intake levels established by authoritative institutions both domestically and internationally [82].

Based on the data obtained, it is roughly estimated that when the intake of a single type of aquatic product per meal increases to 0.6 kg, the amount of heavy metals ingested by adult residents in eastern Guangdong from that type of aquatic product would still be below the tolerable upper intake levels. Therefore, the quality of commercially available aquatic products and food safety for residents in eastern Guangdong is ensured.

**Figure 3:**
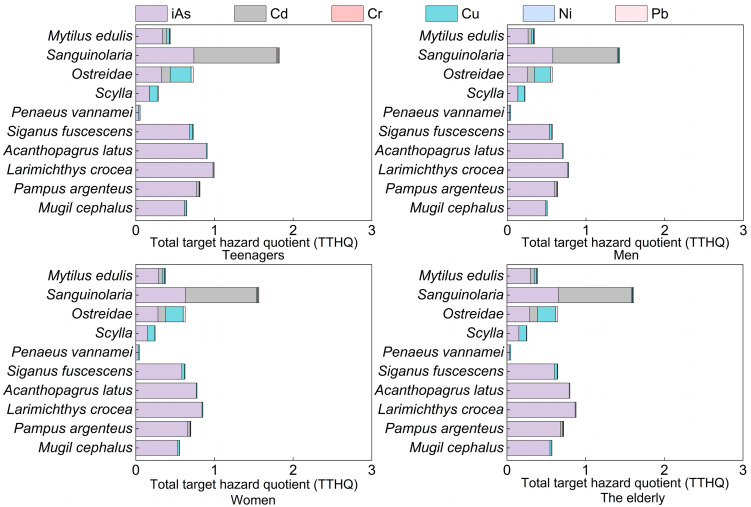
Total target hazard quotient (TTHQ) for edible parts of aquatic products.

It is well established that humans can ingest heavy metals through their diet [83]. Engwa et al. [84] demonstrated that one pathway for heavy metals to enter the human body is through ingestion. Long-term high-dose exposure to heavy metals may pose health risks [21]. Fish come into contact with heavy metals through the food chain, water, and sediments. The presence of toxic heavy metals can impair beneficial nutritional properties [85] and lead to the bioaccumulation and biomagnification of harmful substances in fish and the food chain [86]. Subsequently, these pollutants can be transferred to humans through consumption, potentially causing various adverse health effects such as impaired kidney function, reduced fertility, liver damage, skin cancer, bladder cancer, and even death [87]. Based on the concentrations of heavy metals, the target hazard quotient (THQ) values for different samples were calculated. The results showed that the THQ values for both adults and teenagers were <1 across all samples (Table S6). It indicates that there is no risk to the health of residents, while the THQ value for *Sanguinolaria* sp. was 0.74, suggesting that the concentration of heavy metals in the edible tissues of bivalves from the eastern Guangdong coastal waters poses a relatively low health risk to residents of Shantou. These conclusions suggest that the intake of heavy metals by residents of Shantou is safe when consuming fish reasonably, with no consumption risks observed. Additionally, the TTHQ values for bivalves were all greater than 1 (Figure 3), consistent with the findings of Liu et al. [88] who reported that in Kaozhouyang Bay, an area similarly affected by industrial centers and marine aquaculture, the TTHQ values for metals in bivalves consumed by urban and rural residents were far above the safety threshold (=1), indicating a potential risk of adverse effects. Our study suggests that the consumption of aquatic products by residents of eastern Guangdong is unlikely to pose a health risk. However, if consumption exceeds the average per capita intake, the potential risks of iAs and Cd cannot be ruled out. To minimize the potential adverse health effects of these heavy metals, we recommend limiting the consumption of bivalves, particularly for vulnerable groups such as children and the elderly. Future research on heavy metal bioaccessibility and bioavailability through seafood consumption, as well as arsenic speciation in seafood, will enhance the understanding of the potential health risks associated with toxic heavy metal exposure.

## 4. Conclusions

This study assessed heavy metal pollution in eastern Guangdong aquatic products, finding As, Cd, and Cu levels in bivalves exceeding safety standards, posing potential health risks. This study found heavy metal pollution in all ten types of aquatic products, with bivalves showing particularly high concentrations of As and Cd, some exceeding safety limits. Fish species like *Mugil cephalus* and *Penaeus vannamei* had relatively low pollution levels that met national standards, while certain *Sanguinolaria* sp. exceeded Cd limits and some *Scylla* sp. showed nickel contamination. Heavy metal distribution in fish tissues revealed that the liver is the primary accumulation site, followed by the gills, with lower concentrations in muscle tissue. This suggests that fish detoxify and accumulate metals in the liver. The study also found that carnivorous fish had higher heavy metal concentrations. The assessments of average weekly intake (AWI) and target hazard quotient (THQ) revealed that the THQ values for all samples were below 1, indicating that the heavy metal intake of residents generally falls within safe limits. However, the total target hazard quotient (TTHQ) values for *Sanguinolaria* sp. exceeded 1, suggesting potential health risks associated with their consumption. The study recommends a balanced diet, limiting bivalve consumption, and continued monitoring to ensure food safety. This study provides valuable empirical data on the status of heavy metal pollution in aquatic products from the eastern Guangdong region, highlighting the importance of regular monitoring and risk assessment.

## Figures and Tables

**Table 1:** Frequency of aquatic product ingestion by residents in eastern Guangdong.

Classification	Frequency of Feeding				
	Multiple Times a Week	Once a Week	Once a Month	Six Times a Year	Rarely Consumed
*Mugil cephalus*	12.99%	10.82%	15.58%	19.48%	41.13%
*Pampus argenteus*	8.66%	16.45%	22.94%	21.65%	30.30%
*Larimichthys crocea*	4.76%	13.42%	26.84%	24.68%	30.30%
*Acanthopagrus latus*	14.72%	19.91%	18.61%	15.15%	31.60%
*Siganus fuscescens*	14.72%	14.72%	13.85%	17.75%	38.96%
*Penaeus vannamei*	23.81%	29.87%	23.38%	16.02%	6.93%
*Scylla* sp.	7.36%	9.52%	27.27%	34.63%	21.21%
*Ostreidae* sp.	12.55%	18.61%	22.94%	25.97%	19.91%
*Sanguinolaria* sp.	6.49%	9.96%	17.32%	24.24%	41.99%
*Mytilus edulis*	7.79%	7.36%	22.51%	27.27%	35.06%

**Table 2:** Heavy metal pollution index of aquatic products.

Classification	Latin Name	Heavy Metal Pollution Index (P_i_)					
		iAs	Cd	Cr	Cu	Ni	Pb
Fish	*Mugil cephalus*	1.450	0.000	0.012	0.002	0.000	0.016
	*Pampus argenteus*	1.800	0.041	0.016	0.000	0.016	0.008
	*Larimichthys crocea*	2.300	0.000	0.000	0.001	0.000	0.016
	*Acanthopagrus latus*	2.100	0.000	0.001	0.001	0.000	0.005
	*Siganus fuscescens*	0.320	0.000	0.033	0.004	0.009	0.018
Crustaceans	*Penaeus vannamei*	0.150	0.000	0.000	0.032	0.000	0.004
	*Scylla* sp.	0.768	0.001	0.143	0.163	0.252	0.009
Bivalves	*Ostreidae* sp.	1.600	0.353	0.077	0.344	0.031	0.131
	*Sanguinolaria* sp.	3.600	3.423	0.108	0.018	0.207	0.054
	*Mytilus edulis*	1.640	0.180	0.095	0.042	0.249	0.041

**Table 3:** Average weekly intake of heavy metals in adults and their ratio to PTWI (adults) or UL (weekly).

Group	Aquatic Organism	Latin Name	iAs (1 × 10^−2^)		Cr (1 × 10^−2^)		Cu (1 × 10^−2^)		Cd (1 × 10^−2^)		Pb (1 × 10^−2^)	
			AWI/mg	AWI/UL (%)	AWI/mg	AWI/UL (%)	AWI/mg	AWI/UL (%)	AWI/mg	AWI/UL (%)	AWI/mg	AWI/UL (%)
Teenagers	Pelagic fish	*Mugil cephalus*	0.82	0.56	0.27	7.66	0.49	0.89	0.00	0.00	0.02	1.18
	Midwater fish	*Pampus argenteus*	0.95	0.65	0.23	6.57	0.10	0.18	10.20	16.20	0.01	0.59
		*Larimichthys crocea*	1.26	0.86	0.00	0.00	0.20	0.36	0.00	0.00	0.01	0.59
	Demersal fish	*Acanthopagrus latus*	1.68	1.14	0.02	0.54	0.30	0.55	0.00	0.00	0.01	0.59
		*Siganus fuscescens*	0.90	0.61	0.72	20.57	0.84	1.53	0.00	0.00	0.02	1.18
	Crustaceans	*Penaeus vannamei*	0.41	0.28	0.00	0.00	9.75	17.73	0.00	0.00	0.02	1.18
		*Scylla*	2.03	1.38	0.65	18.69	27.54	50.07	18.37	36.29	0.02	1.18
	Bivalves	*Ostreidae*	4.50	3.06	0.49	14.06	59.34	107.89	100.00	177.51	1.26	74.12
		*Sanguinolaria*	9.20	6.26	0.53	15.14	2.35	4.27	810.23	1417.24	0.37	21.76
		*Mytilus edulis*	5.30	3.61	0.41	11.71	4.64	8.44	46.94	69.39	0.27	15.88
Adults	Pelagic fish	*Mugil cephalus*	1.45	0.99	0.41	11.59	0.82	1.47	0.00	0.00	0.02	1.09
	Midwater fish	*Pampus argenteus*	1.80	1.22	0.30	8.63	0.19	0.34	0.08	16.20	0.01	0.61
		*Larimichthys crocea*	2.30	1.56	0.00	0.00	0.20	0.35	0.00	0.00	0.01	0.41
	Demersal fish	*Acanthopagrus latus*	2.10	1.43	0.03	0.89	0.49	0.88	0.00	0.00	0.01	0.65
		*Siganus fuscescens*	1.60	1.09	1.26	35.88	1.59	2.85	0.00	0.00	0.02	1.25
	Crustaceans	*Penaeus vannamei*	0.75	0.51	0.00	0.00	18.39	32.84	0.00	0.00	0.02	0.94
		*Scylla*	3.84	2.61	1.09	31.17	53.62	95.76	0.18	36.29	0.02	0.98
	Bivalves	*Ostreidae*	8.00	5.44	0.75	21.43	112.38	200.68	0.86	177.51	1.26	72.00
		*Sanguinolaria*	18.00	12.24	0.90	25.69	3.67	6.55	6.94	1417.24	0.37	18.69
		*Mytilus edulis*	8.20	5.58	0.70	20.00	8.36	14.93	0.34	69.39	0.27	15.43
The elderly	Pelagic fish	*Mugil cephalus*	0.71	0.48	0.20	11.82	0.10	0.18	0.00	0.00	0.01	0.59
	Midwater fish	*Pampus argenteus*	0.85	0.58	0.16	9.41	0.09	0.17	8.16	16.20	0.01	0.29
		*Larimichthys crocea*	0.10	0.07	0.00	0.00	0.10	0.18	0.00	0.00	0.01	0.29
	Demersal fish	*Acanthopagrus latus*	1.03	0.70	0.18	10.59	0.24	0.44	0.00	0.00	0.01	0.29
		*Siganus fuscescens*	0.75	0.51	0.59	34.71	0.78	1.42	0.00	0.00	0.01	0.59
	Crustaceans	*Penaeus vannamei*	0.33	0.22	0.00	0.00	9.19	16.70	0.00	0.00	0.01	0.59
		*Scylla*	1.56	1.06	0.49	28.76	2.64	4.80	0.00	36.29	0.01	0.59
	Bivalves	*Ostreidae*	4.00	2.72	0.38	22.06	5.21	9.47	83.68	177.51	0.63	37.06
		*Sanguinolaria*	8.00	5.44	0.43	25.29	1.81	3.29	697.98	1417.24	0.19	10.88
		*Mytilus edulis*	3.58	2.44	0.32	18.82	4.01	7.29	32.65	69.39	0.14	7.94

## Data Availability

The data that support the findings of this study are available from the corresponding author [Z.S.], upon reasonable request.

## Supplementary Materials

The following supporting information can be downloaded at https://www.mdpi.com/article/10.3390/toxics12120881/s1, Figure S1. Correlation between the concentration of heavy metals in edible parts of fish and body length and weight; Figure S2. The trend of heavy metals accumulation in fishes corresponding to the hepatosmatic index; Table S1. ICP-MS/MS and HPLC operating conditions; Table S2. Determination of certified reference material in this study in comparison to the certified values: the certified value is in the bracket; Table S3. Total arsenic and inorganic arsenic determination quality control; Table S4. Quality control for the measurements of heavy metals, including correlation coefficient (R2) of the calibration curve, limit of detection (LOD), limit of quantification (LOQ) and recovery of the certified reference materials; Table S5. Questionnaire survey results on the general demographic information of local residents, including age, gender, and body weight; Table S6. Data from other published studies; Table S7. Target Hazard Quotient (THQ) of different heavy metals in the edible parts of aquatic products. Refs. [12,49,53,54,55,56,57,58,59,60,61,62,63,64,65] are cited in the Supplementary Materials.
