# Supplementary material for: Heavy Metal Distribution in Aquatic Products from Eastern Guangdong and Associated Health Risk Assessment
Source: Toxics. 2024 Dec 2;12(12):881. doi: 10.3390/toxics12120881 (PMC11679869; doi:10.3390/toxics12120881)
Supplement: Supplementary file 1 [file toxics-12-00881-s001.zip › toxics-3343693-supplementary.pdf]

Heavy Metals Distribution in Aquatic Products from Eastern Guangdong and Associated Health Risk Assessment

Jinyan Liu[1, 2,3], You'an Yu[1,2], Zewei Sun[1,2,3*], Keqin Zhang[1,2], Ping Li[1,2], Wenhua Liu[1,2], Ran Bi[1, 2,3]

[1] Guangdong Provincial Key Laboratory of Marine Disaster Prediction and Prevention, and Institute of Marine Sciences, Shantou University, Shantou 515063, China

[2] Institute of Marine Sciences, Shantou University, Shantou 515063, China

[3] Guangdong Engineering Technology Research Center of Offshore Environmental Pollution Control, Shantou 515063, China

* Corresponding authors: zwsun@stu.edu.cn (Zewei Sun)

Contents

Figure S1 Correlation between the concentration of heavy metals in edible parts of fish and body length and weight

Figure S2 The trend of heavy metals accumulation in fishes corresponding to the hepatosmatic index

Table S1 ICP-MS/MS and HPLC operating conditions

Table S2 Determination of certified reference material in this study in comparison to the certified values: the certified value is in the bracket

Table S3 Total arsenic and inorganic arsenic determination quality control

Table S4 Quality control for the measurements of heavy metals, including correlation coefficient (R2) of the calibration curve, limit of detection (LOD), limit of quantification (LOQ) and recovery of the certified reference materials

Table S5 Questionnaire survey results on the general demographic information of local residents, including age, gender, and body weight

Table S6 Data from other published studies

Table S7 Target Hazard Quotient (THQ) of different heavy metals in the edible parts of aquatic products

Figure S1 Correlation between the concentration of heavy metals in edible parts of fish and body length and weight

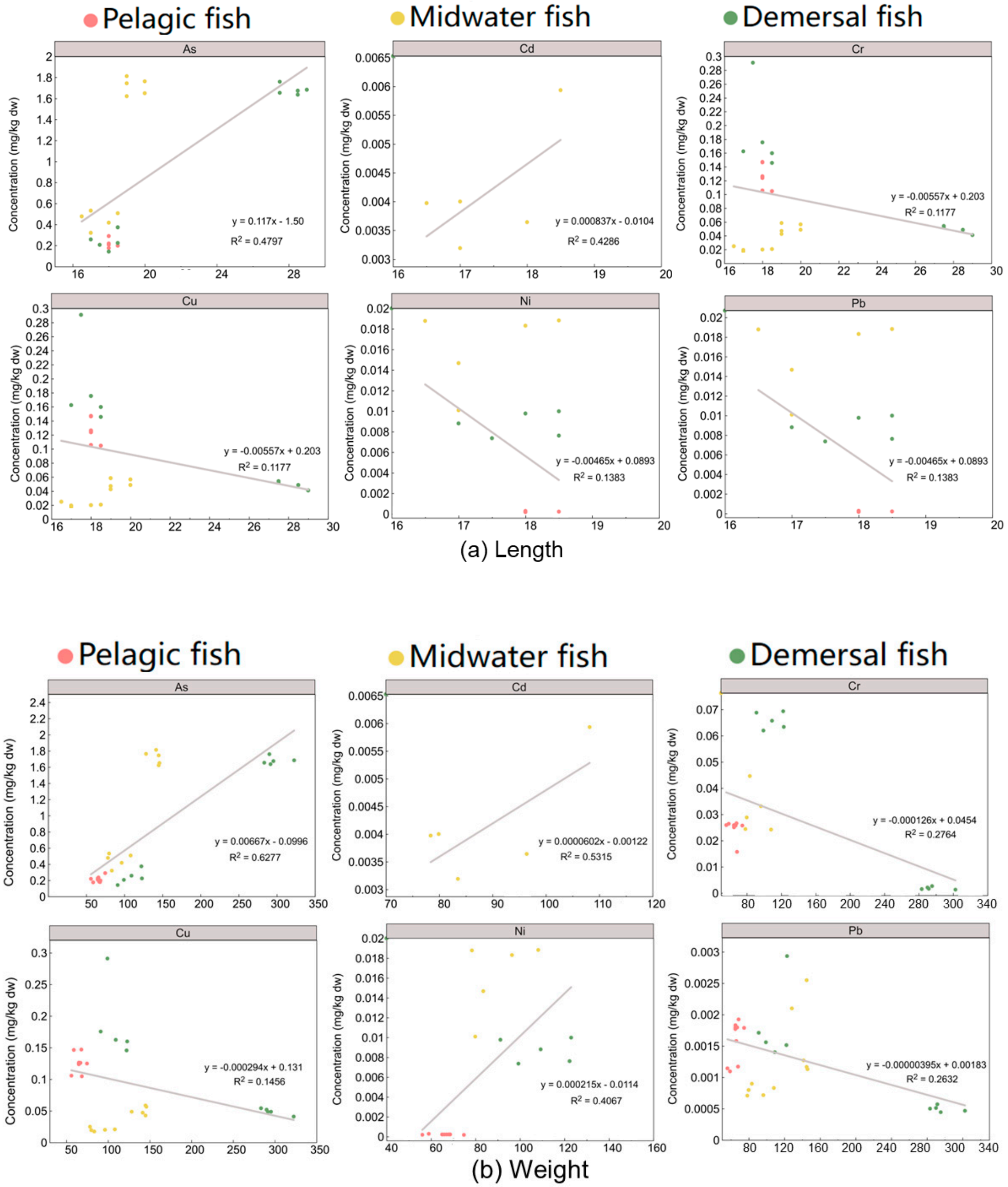

Figure S2 The trend of heavy metals accumulation in fishes corresponding to the hepatosmatic index

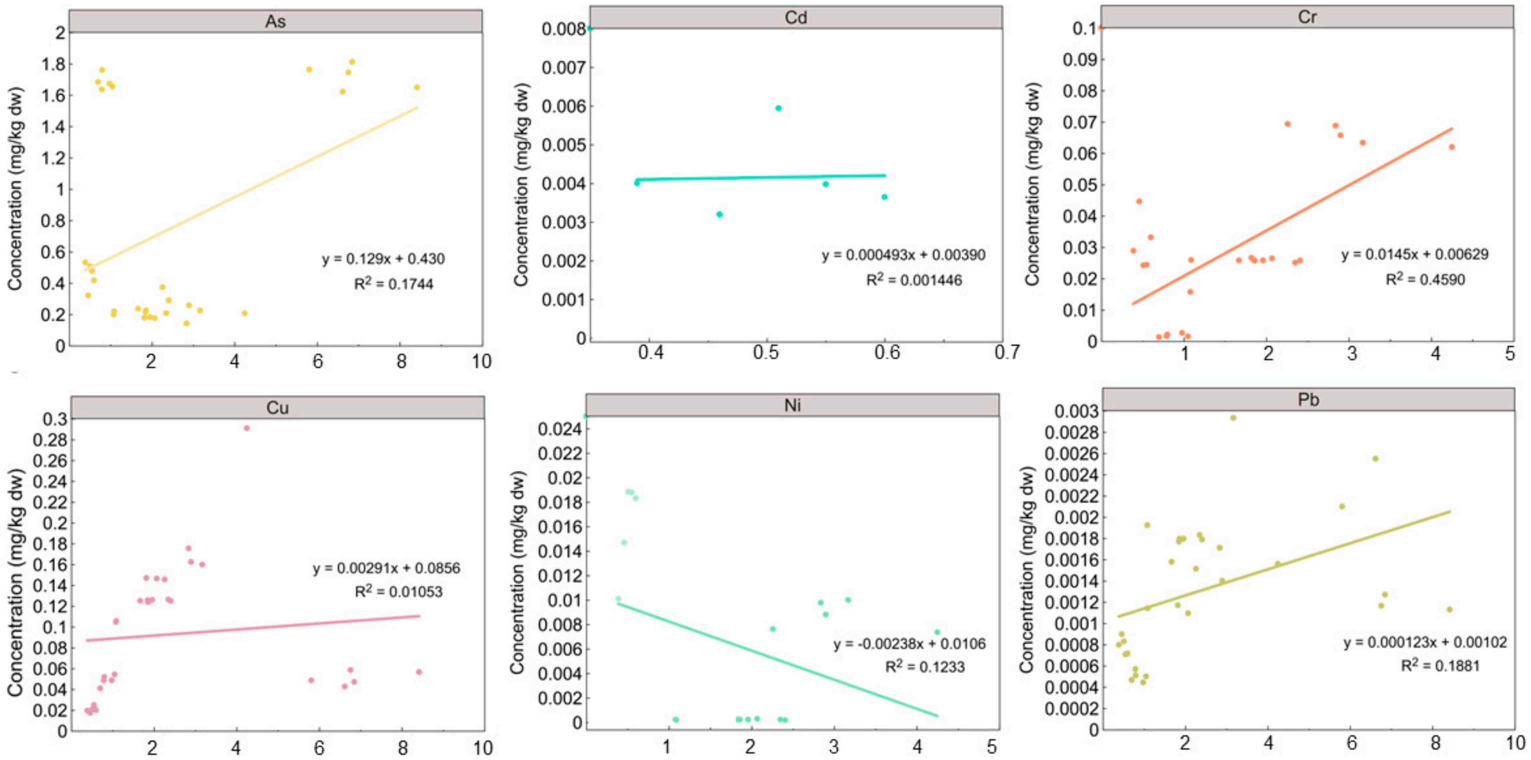

Table S1 ICP-MS/MS and HPLC operating conditions

	Operating conditions
**ICP-MS**	
RF power	1550W
Cones	Ni
Plasma, nebulizer and makeup gas flows	14, 1 and 0.8 L/min
Spray chamber temperature	2 °C
Reaction cell gas	O_2
Monitored isotopes (Q1→Q2)	As (*m/z* 75 → *m/z* 91)
Integration time	0.1 s[a], 0.2 s[b]
Replicates per analysis	6[a], 1[b]
Peak pattern	1 point per mass
**HPLC**	
Guard Column	Dionex IonPac AG7
Analytical Column	Dionex IonPac AG7
Mobile phase	(A) 2.5 mM $(NH_4)_2CO_3$
	(B) 125 mM $(NH_4)_2CO_3$
Gradient profile	0-4.5 min (100% A)
	4.6-28.5 min (100% B)
	28.6-30 min (100% A)
Flow rate	0.85 mL/min
Autosampler temperature	4 °C
Injection volume	20 µL
Column temperature	Ambient

[a] for heavy metal determination by ICP-MS/MS
[b] for iAs analysis by HPLC-ICP-MS/MS

Table S2 Determination of certified reference material in this study in comparison to the certified values: the certified value is in the bracket

	As mg/kg	Cd mg/kg	Cr mg/kg	Cu mg/kg	Ni mg/kg	Pb mg/kg
GBW10024 (certified value)	3.82±0.37 (3.6±0.6)	1.21±0.01 (1.06±0.1)	0.26±0.002 (0.28±0.07)	1.16±0.01 (1.34±0.18)	0.25±0 (0.29±0.01)	0.10±0.08 (0.120)
SRM1566b (certified value)	7.91±0.02 (7.65±0.65)	2.8±0.05 (2.48±0.08)	0.48±0.004 N/A	64.671±0.242 (71.6±1.6)	1.103±0.017 (1.04±0.09)	0.322±0.003 (0.308±0.009)
BCR-627 (certified value)	5.03±0.03 (4.8±0.3)	N/A	N/A	N/A	N/A	N/A
Recovery	103-106%	112-114%	92.9-103.4%	86.6-90.3%	86.2-106.1%	83.3-104.5%

N/A = No certified data available

Table S3 Total arsenic and inorganic arsenic determination quality control

	Total As<br>mg/kg	iAs<br>mg/kg	Extraction Efficiency<br>(%)
NMIJ-7405b<br>(certified value)	50.1±1.2<br>(49.5±1.0)	17.9±0.7<br>(24.4±0.7)	70
Recovery	107.1%	104.8%*	

*inorganic arsenic concentration considered extraction efficiency of 70%.

Table S4 Quality control for the measurements of heavy metals, including correlation coefficient (R2) of the calibration curve, limit of detection (LOD), limit of quantification (LOQ) and recovery of the certified reference materials

**Heavy metal**	**R^2**	**LOD (μg/L)**	**LOQ (μg/L)**
As	0.9987	0.0108	0.0324
Cd	0.9923	0.00068	0.0020
Cr	0.9994	0.0022	0.0066
Cu	0.9996	0.0229	0.0687
Ni	0.9978	0.0376	0.1128
Pb	0.9995	0.00087	0.0026

Table S5 Questionnaire survey results on the general demographic information of local residents, including age, gender, and body weight

Gender	Age	Body weight (kg)	Number
Male	≤ 20	≤ 45	28
		45 – 60	13
		60 – 75	8
		75 – 90	2
		≥ 90	0
	20 – 60	≤ 45	18
		45 – 60	30
		60 – 75	22
		75 – 90	9
		≥ 90	1
	≥ 60	≤ 45	8
		45 – 60	11
		60 – 75	4
		75 – 90	2
		≥ 90	0
Female	≤ 20	≤ 45	14
		45 – 60	4
		60 – 75	1
		75 – 90	0
		≥ 90	0
	20 – 60	≤ 45	16
		45 – 60	17
		60 – 75	9
		75 – 90	3
		≥ 90	0
	≥ 60	≤ 45	4
		45 – 60	5
		60 – 75	2
		75 – 90	0
		≥ 90	0
Total			231

Table S6 Data from other published studies

Classification	Latin Name	As Conc. (mg/kg)	Cd Conc. (mg/kg)	Cr Conc. (mg/kg)	Cu Conc. (mg/kg)	Ni Conc. (mg/kg)	Pb Conc. (mg/kg)	Location	Tissue	Ref.
Fish		0.90 ± 0.10 ww	0.02 ± 0.002 ww				0.07 ± 0.01 ww	Varna Lake Nesebar	muscle	(Stancheva et al., 2013)
		0.38 ± 0.02 ww	0.03 ± 0.003 ww				0.08 ± 0.02 ww		gill	
	Mugil cephalus		17.44 ± 7.96 ww		32.98 ± 3.13 ww		6.75 ± 3.25 ww	Woji, southern Nigeria	muscle	(Ihunwo et al., 2020)
			38.43 ± 14.19 ww	0.73 ± 1.03 ww	51.44 ± 12.57 ww		22.43 ± 5.16 ww		gill	
			17.99 ± 2.29 ww	0.57 ± 0.36 ww	16.03 ± 8.66 ww		2.58 ± 0.66 ww		liver	
			0.02 ± 0.021 ww	0.73 ± 1.03 ww		0.05 ± 0.05 ww	1.39 ± 1.43 ww	Coasts of Tanzania	muscle	(Mwakalapa et al., 2019)
			0.01 ± 0.01 ww		0.90 ± 0.98 ww	0.06 ± 0.06 ww	3.46 ± 3.34 ww		liver	
	Pampus argenteus		0.031 ± 0.02 ww	0.233 ± 0.01 ww	0.091 ± 0.059 ww		0.277 ±	Yancheng inshore in Jiangsu	muscle	(Chen et al., 2023)

							0.056 ww			
	Larimichthys crocea	0.12 ± 0.10 ww	0.065 ± 0.001 ww		2.4 ± 0.10 ww		0.035 ± 0.10 ww	Dachen Islands, East China Sea	muscle	(Huang et al., 2022)
		0.22 ± 0.30 ww	0.067 ± 0.001 ww		2.7 ± 0.10 ww		0.35 ± 0.10 ww		gill	
		0.15 ± 0.10 ww	0.071 ± 0.002 ww		17 ± 0.10 ww		0.1 ± 0.10 ww		liver	
	Acanthopagrus latus	0.0072 ± 0.0006 ww	0.0084 ± 0.0015 ww	0.19 ± 0.0094 ww			0.016 ± 0.001 ww	South of the Khuzestan province, Iran	muscle	(Norouzi, 2020)
	Siganus fuscescens	0.48 ± 0.03 ww	0.002 ± 0.001 ww	4.36 ± 1.60 ww	0.28 ± 0.02 ww		0.05 ± 0.018 ww	Hainan Island in China	muscle	(Liu et al., 2015)
		1.07 ± 0.007 ww	0.10 ± 0.06 ww	3.31 ± 0.41 ww	0.9 ± 0.24 ww		0.22 ± 0.03 ww		gill	
		1 ± 0.07 ww	0.16 ± 0.08 ww	1.52 ± 0.06 ww	2.27 ± 0.67 ww		0.11 ± 0.02 ww		liver	
Crustaceans	*Penaeus vannamei*			20.86 ± 5.27 dw	24.26 ± 8.36 dw			Zhanjiang Harbour Bay	muscle	(Wu and Yang, 2011)

	3.30 ± 1.29 dw	18.91 ± 5.50 dw	126.42 ± 90.92 dw				liver	
			19.60 ± 1.0 dw	1.0 ± 0.1 dw		Northwest Coast of M6xico	muscle	(Liu et al., 2021)
	0.13 ± 0.001 ww		0.41 ± 0.05 ww		0.09 ± 0.01 ww	Punnakayal in Tuticorin, Southeast Coast of India	muscle	
	0.14 ± 0.001 ww		3.63 ± 0.57 ww		0.19 ± 0.01 ww		gill	
	0.32 ± 0.001 ww		0.66 ± 0.07 ww		0.25 ± 0.02 ww	Harbour Beach in Tuticorin, Southeast Coast of India	muscle	(Yogeshwaran et al., 2020)
Scylla serrata	0.18 ± 0.002 ww		5.27 ± 1.0 ww		0.55 ± 0.03 ww		gill	
	0.64 ± 0.002 ww		0.86 ± 0.03 ww		0.59 ± 0.06 ww	Threspuram in Tuticorin, Southeast Coast of India	muscle	
	0.23 ± 0.05 ww		10.6 ± 1.02 ww		0.72 ± 0.02 ww		gill	
	0.23 ± 0.061 ww	0.337 ± 0.061 ww	5.494 ± 0.430 ww		0.307 ± 0.061 ww	East Java Estuaries of Indonesia	muscle	(Soegianto et al., 2022)

		1.42 ± 1.61 ww		0.09 ± 0.15 ww	4.06 ± 2.30 ww		0.02 ± 0.05 ww	Setiu Wetlands in Malaysia	muscle	(Razali et al., 2024)
		1.16 ± 0.87 ww	0.03 ± 0.02 ww	9.26 ± 2.56 ww	45.51 ± 16.0 ww	0.47 ± 0.27 ww	0.82 ± 0.20 ww	Kuala Sepetang in Malaysia	muscle	
Bivalves	*Ostreidae*		10.51 ± 3.77 ww		100.59 ± 26.75 ww		2.79 ± 1.12 ww	North-central coast of Sinaloa	muscle	(Muñoz Sevilla et al., 2017)
	Sanguinolaria acuminata				15.72 ± 5.0 ww		4.27 ± 0.12 ww	coastal waters of India	muscle	(Sarkar et al., 2008)
			6.74 ± 0.2 ww		80.09 ± 11.0 ww		42.53 ± 12 ww		gill	
	Mytilus edulis	15.31 ± 0.02 dw	0.52 ± 0.01 dw				0.7 ± 0.1 dw	western coast of Bergen, Norway	muscle	(Gomez-Delgado et al., 2023)

Table S7 Target Hazard Quotient (THQ) of different heavy metals in the edible parts of aquatic products

Population	Species	iAs	Cd	Cr	Cu	Ni	Pb
Teenagers	*Mugil cephalus*	0.61845	0	1.35E-04	0.02591	8.88E-05	0.00332
	Pampus argenteus	0.76773	0.03538	1.76E-04	0.00435	0.00668	0.00168
	Larimichthys crocea	0.98099	0	0	0.01079	0	0.0035
	Acanthopagrus latus	0.89569	0	1.04E-05	0.0104	0	0.00106
	Siganus fuscescens	0.68243	0	3.74E-04	0.03983	0.0036	0.00389
	Penaeus vannamei	0.03333	0	0	0.0216	0	2.76E-04
	Scylla	0.17067	3.10E-04	1.02E-04	0.10875	0.00653	6.07E-04
	Ostreidae	0.3283	0.10859	6.29E-05	0.26448	9.22E-04	0.03029
	Sanguinolaria	0.73867	1.05346	8.90E-05	0.01415	0.00618	0.01245
	Mytilus edulis	0.3365	0.05545	7.79E-05	0.03247	0.00745	0.00947
Men	*Mugil cephalus*	0.48401	0	1.06E-04	0.02028	6.95E-05	0.0026
	Pampus argenteus	0.60083	0.02769	1.38E-04	0.00341	0.00523	0.00132
	Larimichthys crocea	0.76773	0	0	0.00845	0	0.00274
	Acanthopagrus latus	0.70097	0	8.14E-06	0.00814	0	8.31E-04
	Siganus fuscescens	0.53408	0	2.93E-04	0.03117	0.00282	0.00304
	Penaeus vannamei	0.02609	0	0	0.01691	0	2.16E-04
	Scylla	0.13357	2.42E-04	7.98E-05	0.08511	0.00511	4.75E-04
	Ostreidae	0.25693	0.08498	4.92E-05	0.20698	7.21E-04	0.02371
	Sanguinolaria	0.57809	0.82444	6.96E-05	0.01108	0.00484	0.00974
	Mytilus edulis	0.26335	0.0434	6.10E-05	0.02541	0.00583	0.00741

Women	*Mugil cephalus*	0.5301	0	1.16E-04	0.02221	7.61E-05	0.00284
	Pampus argenteus	0.65806	0.03032	1.51E-04	0.00373	0.00573	0.00144
	Larimichthys crocea	0.84085	0	0	0.00925	0	0.003
	Acanthopagrus latus	0.76773	0	8.92E-06	0.00892	0	9.10E-04
	Siganus fuscescens	0.58494	0	3.21E-04	0.03414	0.00309	0.00333
	Penaeus vannamei	0.02857	0	0	0.01852	0	2.36E-04
	Scylla	0.14629	2.66E-04	8.74E-05	0.09321	0.0056	5.21E-04
	Ostreidae	0.2814	0.09308	5.39E-05	0.22669	7.90E-04	0.02596
	Sanguinolaria	0.63314	0.90296	7.63E-05	0.01213	0.0053	0.01067
	Mytilus edulis	0.28843	0.04753	6.68E-05	0.02783	0.00639	0.00811
The elderly	*Mugil cephalus*	0.54569	0	1.19E-04	0.02286	7.83E-05	0.00293
	Pampus argenteus	0.67741	0.03122	1.56E-04	0.00384	0.00589	0.00149
	Larimichthys crocea	0.86558	0	0	0.00952	0	0.00309
	Acanthopagrus latus	0.79031	0	9.18E-06	0.00918	0	9.36E-04
	Siganus fuscescens	0.60214	0	3.30E-04	0.03514	0.00318	0.00343
	Penaeus vannamei	0.02941	0	0	0.01906	0	2.43E-04
	Scylla	0.15059	2.73E-04	9.00E-05	0.09595	0.00576	5.36E-04
	Ostreidae	0.28967	0.09582	5.55E-05	0.23336	8.13E-04	0.02673
	Sanguinolaria	0.65176	0.92952	7.85E-05	0.01249	0.00545	0.01098
	Mytilus edulis	0.29692	0.04893	6.87E-05	0.02865	0.00658	0.00835

Reference

Chen, Y., Chen, X., Jiang, R., Li, S., Ma, X., Sun, Y., Zhang, T. & Feng, Z. 2023. Bioaccumulation characteristics of typical pollutants in seafood from coastal waters of Jiangsu, China. *Continental Shelf Research,* 263**,** 105030.

Gomez-Delgado, A. I., Tibon, J., Silva, M. S., Lundebye, A.-K., Agüera, A., Rasinger, J. D., Strohmeier, T. & Sele, V. 2023. Seasonal variations in mercury, cadmium, lead and arsenic species in Norwegian blue mussels (Mytilus edulis L.)–Assessing the influence of biological and environmental factors. *Journal of Trace Elements in Medicine and Biology,* 76**,** 127110.

Huang, H., Li, Y., Zheng, X., Wang, Z., Wang, Z. & Cheng, X. 2022. Nutritional value and bioaccumulation of heavy metals in nine commercial fish species from Dachen Fishing Ground, East China Sea. *Scientific Reports,* 12**,** 6927.

Ihunwo, O. C., Dibofori-Orji, A. N., Olowu, C. & Ibezim-Ezeani, M. U. 2020. Distribution and risk assessment of some heavy metals in surface water, sediment and grey mullet (Mugil cephalus) from contaminated creek in Woji, southern Nigeria. *Marine Pollution Bulletin,* 154**,** 111042.

Liu, J.-L., Xu, X.-R., Ding, Z.-H., Peng, J.-X., Jin, M.-H., Wang, Y.-S., Hong, Y.-G. & Yue, W.-Z. 2015. Heavy metals in wild marine fish from South China Sea: levels, tissue-and species-specific accumulation and potential risk to humans. *Ecotoxicology,* 24**,** 1583-1592.

Liu, S., Chen, H., Wang, J., Su, L., Wang, X., Zhu, J. & Lan, W. 2021. The distribution of microplastics in water, sediment, and fish of the Dafeng River, a remote river in China. *Ecotoxicology and environmental safety,* 228**,** 113009.

Muñoz Sevilla, N. P., Villanueva-Fonseca, B. P., Góngora-Gómez, A. M., García-Ulloa, M., Domínguez-Orozco, A. L., Ortega-Izaguirre, R. & Campos Villegas, L. E. 2017. Heavy metal concentrations in diploid and triploid oysters (Crassostrea gigas) from three farms on the north-central coast of Sinaloa, Mexico. *Environmental monitoring and assessment,* 189**,** 1-10.

Mwakalapa, E. B., Simukoko, C. K., Mmochi, A. J., Mdegela, R. H., Berg, V., Müller, M. H. B., Lyche, J. L. & Polder, A. 2019. Heavy metals in farmed and wild milkfish (Chanos chanos) and wild mullet (Mugil cephalus) along the coasts of Tanzania and associated health risk for humans and fish. *Chemosphere,* 224**,** 176-186.

Norouzi, M. 2020. Evaluating the accumulation and consumption hazard risk of heavy metals in the fish muscles of species living in the waters of the Persian Gulf, Iran. *Pollution,* 6**,** 849-862.

Razali, N. S. M., Ikhwanuddin, M., Maulidiani, M., Gooderham, N. J., Alam, M. & Abd Kadir, N. H. 2024. Ecotoxicological impact of heavy metals on wild mud crabs (Scylla olivacea) in Malaysia: An integrative approach of omics, molecular docking and human risk assessment. *Science of The Total Environment,* 946**,** 174210.

Sarkar, S. K., Cabral, H., Chatterjee, M., Cardoso, I., Bhattacharya, A. K., Satpathy, K. K. & Alam, M. A. 2008. Biomonitoring of heavy metals using the bivalve molluscs in Sunderban mangrove wetland, northeast coast of Bay of Bengal (India): possible risks to human health. *CLEAN–Soil, Air, Water,* 36**,** 187-194.

Soegianto, A., Wahyuni, H. I., Yulianto, B. & Abd Manaf, L. 2022. Health risk assessment of metals in mud crab (Scylla serrata) from the East Java Estuaries of Indonesia. *Environmental Toxicology and Pharmacology,* 90**,** 103810.

Stancheva, M., Makedonski, L. & Petrova, E. 2013. Determination of heavy metals (Pb, Cd, As and Hg) in Black Sea grey mullet (Mugil cephalus). *Bulgarian Journal of Agricultural Science,* 19**,** 30-34.

Wu, X.-Y. & Yang, Y.-F. 2011. Heavy metal (Pb, Co, Cd, Cr, Cu, Fe, Mn and Zn) concentrations in harvest-size white shrimp Litopenaeus vannamei tissues from aquaculture and wild source. *Journal of Food Composition and Analysis,* 24**,** 62-65.

Yogeshwaran, A., Gayathiri, K., Muralisankar, T., Gayathri, V., Monica, J. I., Rajaram, R., Marimuthu, K. & Bhavan, P. S. 2020. Bioaccumulation of heavy metals, antioxidants, and metabolic enzymes in the crab Scylla serrata from different regions of Tuticorin, Southeast Coast of India. *Marine pollution bulletin,* 158**,** 111443.
